# Linking Positive Psychology and Intercultural Competence by Movies: Evidence From Brunei and Romania

**DOI:** 10.3389/fpsyg.2021.750904

**Published:** 2021-10-19

**Authors:** Daniela Popa, Florin Nechita, Yong Liu, Shirley Wei Lee Chin

**Affiliations:** ^1^Department of Psychology, Education and Teacher Training, Transilvania University of Brasov, Brasov, Romania; ^2^Department of Social and Communication Sciences, Transilvania University of Brasov, Brasov, Romania; ^3^Faculty of Arts and Social Sciences, Design and Creative Industries Programme, Universiti Brunei Darussalam, Bandar Seri Begawan, Brunei; ^4^Faculty of Arts and Social Sciences, Geography, Environment and Development Programmme, Universiti Brunei Darussalam, Bandar Seri Begawan, Brunei

**Keywords:** wellbeing, intercultural awareness, PERMA model, cinematic storytelling, intercultural sensitivity

## Abstract

Cultural consumption provides numerous benefits for individuals, especially for younger generations. Imaginary travel narratives can shape people’s perceptions about other cultures thus are useful tools for developing intercultural competences. On the other hand, positive psychology provides an approach to understand different aspects of students/youngsters’ wellbeing. This study investigates the wellbeing associated with learning the meanings of being different and growing in emotional resilience, flexibility, and openness to other cultures through movies. The positive psychology approach was used to examine the benefits of movie consumption in order to investigate the activation of five domains of wellbeing: positive emotions, engagement, relationships, meaning, and accomplishment (PERMA). The research methodology consisted of the experiment and questionnaire survey. The students’ scores on the wellbeing and intercultural competences were measured before and after the intervention. The intervention consisted of sessions of watching two example movies, Eat Pray Love and Hotel Transylvania 2, and subsequent group discussions about the movies’ respective messages. The participants’ group was formed by 236 university students from Brunei and Romania, ages between 18 and 49years old. The results showed an increase in students’ openness to other cultures and across some of the wellbeing PERMA dimensions. The study makes a theoretical contribution by connecting positive psychology and the intercultural competence constructs and the influences of movies.

## Introduction

Functioning effectively in cross-cultural environments is one of the core cross-cutting competencies in a highly globalized world with teams made up of highly diverse individuals (Akdere et al., 2021). Therefore, students need to be trained to develop a high level of intercultural tolerance in order to integrate into society and increase their employability, an important indicator of accomplishment for most universities. The ability to cooperate, that is to work in multi-ethnic, multinational teams, to be open to new working environments is one of the crucial aspects that employers consider in addition to basic training in a field. In the face of this increase in cultural and socioeconomic diversity, researchers have come up with multiple solutions to implement intercultural competency training programs for both teachers and students (Kohli Bagwe and Haskollar, 2020; Peng et al., 2020; Romijn et al., 2021). Some of these refer to measuring intercultural competence before and after completing a university course (Snodgrass et al., 2018), after a semester of educational exchange experience (Dervin et al., 2020), others involve the use of virtual reality technology to develop intercultural competence (Akdere et al., 2021) and, the ones that caught our attention, are those that use movies as a support in developing intercultural competence (Yue, 2019).

The idea of using film as an authentic teaching medium is not new (Chaya and Inpin, 2020), but it is certainly appropriate for a generation of students with a predominantly visual style of information accumulation. Although the primary intention of most movie producers is not to induce visitors to the filmed locations, the natural attractions, lifestyle, and cultural experiences featured in films do play an impact in influencing viewers awareness (Bolan et al., 2011). With the evolution of media in our lives, movies and television shows have become our mainstream entertainment exposing us to visualized images and culture, thus creating awareness and openness of different cultures through movies. According to Spears et al. (2013), how the location is featured in a movie and how the cultural image is portrayed both affect the viewers’ perception and engagement toward the filming destination. A movie may have the ability to attract tourists to a particular location (Leotta, 2011; Waade, 2016; Chin and Liu, 2018), it also often has embedded cultural content that has implications in our acceptance of cultures (Jane, 2020). Some films shot in a particular location can provide a consistent promise of possibly profound life-changing experiences for potential tourists (Frost, 2010).

Films can also be used in a discreet pedagogy of exemplary acts. Films have often been used as part of therapeutic interventions (Hesley and Hesley, 2001), creating a new niche called cinematherapy. It is well known that today, increasing pressure from school activities translated into high levels of mental illness and stress among university students (Pedrelli et al., 2015; Evans et al., 2018), as some studies reported lower levels of wellbeing for this category compared with the general population (Stewart-Brown et al., 2000). The higher incidence of depression symptoms among university students compared with the general population was reported by Ibrahim et al. (2013), as well as an increasing level of anxiety for first year students (Stallman, 2010). Therefore, this comparative study attempts to investigate the connection between student wellbeing and their intercultural competence constructs through the influence of popular movies with a narrative of traveling to multiple destinations, making both theoretical and practical contribution to the field of positive psychology research.

### Intercultural Competence

The concept of intercultural competence has been intensively debated by researchers and has a rich theoretical foundation. Recent studies show the existence of more than 300 theoretical constructs and terms equivalent to intercultural competence (Spitzberg and Changnon, 2009). In this research, we use the definition of intercultural competence proposed by Bennett (2008), which includes “a set of cognitive, affective, and behavioral skills and characteristics that support effective and appropriate interaction in a variety of cultural contexts” (p. 3). This definition emphasizes the components of competence, knowledge, skills, and attitudes that are based on diversity values.

The theoretical basis of intercultural competence is grounded in the Developmental Model of Intercultural Sensitivity (DMIS) (Bennett, 1986). The developmental model proposed by the theoretical perspective of intercultural competence has its main directions the acquisition by individuals of a global, international perspective of identity and the development of harmonious relationships with people from different cultures (Armstrong, 2020). A high level of development of this competence allows the individual to select appropriate behavior, be more empathetic (Meleady et al., 2020), minimize prejudice, and ethnocentrism (Gregersen-Hermans, 2017), have a higher level of tolerance for ambiguity (Romijn et al., 2021), manage stress more easily (Schwarzenthal et al., 2020), and communicate easily in cross-cultural environments (Stier, 2009). Also, when conceptualizing this term, we need to take into account the fact that today most individuals belong to diverse groups (ethnic, religious, national, occupational, etc.) thus have diverse socio-cultural identities (Barrett, 2018).

From a cognitive behavioral paradigm perspective, an interesting construct within intercultural competence is cultural intelligence (Li, 2020). It focuses on an individual’s ability to adapt efficiently to cultural settings in which people think and behave differently from the person’s culture (Presbitero, 2020). Recent studies highlight that individuals with high levels of cultural intelligence tend to perceive the settings more complexly (Hu et al., 2020). The two theoretical perspectives are included in this research to deepen the connections between these constructs and to uncover new measurement possibilities.

The clear conceptual delineation of “intercultural competence” is still an under-explored aspect in the literature. Thus, often the concept of intercultural competence is overlapped over terms, such as “intercultural sensitivity, cross-cultural effectiveness, intercultural skills, cross-cultural adaptation, global competence, multicultural competence, cross-cultural relations, cultural proficiency, intercultural agility, and even the misnomer cultural intelligence” (Bennett, 2015, p. 483). Intercultural sensitivity is a central concept in the model developed by Bennett, representing an individual’s ability to adapt with cultural differences. Cultural intelligence is defined as “the ability to function in intercultural contexts” (Ang et al., 2015, p. 273). These concepts, although seeming to denote the same reality, actually have subtle demarcations. Thus, intercultural sensitivity refers more to being aware of and understanding cultural differences, and cultural intelligence emphasizes more the perspective of competence in action, what makes individuals to operate effectively in varied cultural contexts. From a complementary perspective, we have chosen to include both “intercultural sensitivity” and “cultural intelligence” as facets of intercultural competence.

### PERMA Students’ Wellbeing

The outbreak of the COVID-19 pandemic and research signals about the negative effects on student mental wellbeing makes relevant all the solutions dealing with this issue, and wellbeing programs from positive psychology perspective being one of these (Morgan and Simmons, 2021). Schools and universities need to have a strategic approach over the use of positive psychology in areas like teacher and school leadership and other tools needed to create a positive environment for students, and Seligman (2011) PERMA model being considered a potential useful framework for fostering positive education in those institutions aiming at improving academic engagement and achieving results (Waters, 2011). The reasons for including positive education in higher education institutions are the encouraging effects on life satisfaction, learning process and creativity, lowering depression, and enhancing civic participation of the students (Seligman et al., 2009; Kern et al., 2015; Wingert et al., 2020). Houghton and Anderson (2017) go further by suggesting that wellbeing should be embedded into the curriculum content.

Seligman (2011) PERMA model was used in many contexts in order to measure wellbeing within educational frameworks. Research into positive education is not related only to formal teaching settings, Oades et al. (2011) recommended that the whole university should be included and gave examples of activities structured on all five PERMA dimensions and oriented toward five key aspects of university life: classroom, social, local community, faculty residential administration, and residential. For local community activities, the authors suggested movie screenings with positive psychological content.

Kern et al. (2015) concluded that measuring subjective perspectives of wellbeing across multiple domains will successfully promote student wellbeing. The increased concern over students’ wellbeing translated into positive education interventions as wellbeing programs addressing this target group with minimal resources and low stigma (Young et al., 2020). The aims of intervention programs are offering to the youth means to attain the skills to achieve greater versions of themselves, something not currently on offer in most academic institutions (Lambert et al., 2019).

The PERMA-Profiler (Butler and Kern, 2016) was used in university contexts for measuring effects of a character strength (Smith et al., 2021), the role of intergenerational program in providing positive experiences and interactions for students (Gray et al., 2020), relationship between affective wellbeing and academic self-efficacy and performance (Cobo-Rendón et al., 2020), examinations of student veterans wellbeing (Umucu et al., 2018a,b, 2019), effects of the patience training program on patience and wellbeing (Bülbül and Izgar, 2018), the relationships between social networking tools and wellbeing (Zhou and Zhang, 2019), and effects of mindfulness-based strengths practice on wellbeing (Wingert et al., 2020).

### Movies Influence in our Lives

Using DRAMMA and PERMA model of wellbeing, Laing and Frost (2017) focus their study on contemporary non-fiction books – some of these books have been adapted into popular films – written by women about their travel experiences in Italy and “examine the Italian transformative journey as a travel trope… omnipresent in women’s travel narratives” that affect “well-being on a broad level” (pp. 116–117). Laing and Frost (2017) further suggest future study could consider whether the trope of transformation also resonates with men visiting Italy in both literary and cinematic contexts (p. 118).

In a matter of fact, Niemiec (2008); 2009, 2010a, 2010b, 2011, 2014, 2015, 2020 has long suggested and discussed approaching movies as character strength-based interventions for both researchers and practitioners in positive psychology field. Niemiec (2009,2010a,b, 2011) and Niemiec and Bretherton (2015) reviewed popular movies, such as *Twilight* (Hardwicke, 2008), *Invictus* (Eastwood, 2009), *Alice in Wonderland* (Burton, 2010), *Happy* (Belic et al., 2012) and *Frozen* (Buck et al., 2014), through the positive psychology lens and elaborated how to carry out the science-based, positive interventions via the use of popular films in the psychotherapy process. Such interventions are based on the *via* classification of 24 strengths and six virtues (Peterson and Seligman, 2004) that have been found universal across cultures and nations (Park et al., 2006). Niemiec and Wedding (2014) claimed “cinema is not restricted to one country or group of people. Therefore, movies are a commentary on more than society – they inform us about the human condition” (p. 3). They went on to associate cinematic storytelling and characters with positive psychology research: “Positive psychology theories, virtues, and strengths lie in the film’s subtext. They emerge as powerful themes and motifs, and as qualities within the characters but beneath the storyline” (p. 3). “Positive psychology grows out of robust research on subjective well-being and character strengths, and it links with humanistic psychology and other avenues of inquiry that emphasize wellness” (p. 7). In particular, the protagonist Elizabeth “Liz” Gilbert in *Eat Pray Love* (Murphy, 2010) from the perspective of PERMA model, “can be viewed as her personal training in each of these three areas (positive emotions, engagement and meaning). She develops pleasure through enjoying the food and wine in Italy, engagement through her meditation training in India, and meaning in the development of purpose and a loving relationship in Bali” (Niemiec and Wedding, 2014, p. 335). Here, Liz’s “loving relationship” with her new boyfriend Felipe in Bali and the happy ending of their reunion on the dock and then sailing on the sea embody the other two components of PERMA model – positive relationships and accomplishment. Not only because the spiritual guru Ketut tells Liz: “Sometimes, to lose balance to love is part of living a balanced life,” so that she realizes the relationship with Felipe is a positive “crossover” for her to “living a balanced life”; but also because, by so doing, Liz eventually accomplishes her truth-searching and self-discovery journey. No wonder *EPL* received the Positive Psychology Film Awards, in the category of Authentic Happiness Theory, given yearly by Niemiec (p. 380).

In *Hotel Transylvania 2 (HT2)*, PERMA model is also reflected, through a diverged dichotomy, in terms of how to train the 5years old Dennis (ovitch), who is fathered by a human Johnny and mothered by a vampire Mavis, to become a human or a vampire. Mavis considers the former way to raise up Dennis as a human by moving to California where Johnny grew up, while Mavis’s father Drac wants his grandson to stay in Transylvania and become a “fanger.” Both Mavis and Drac attempt to bring a positive future, strength, and happiness to Dennis, but only through their respective engagement. Drac even encourages Johnny to bring Mavis to visit his parents in California, but his real intention is to let Johnny keep Mavis distracted during her visit, so that she will not move. At the same time, Drac summons a group of monster friends to help him train Dennis to become a vampire at a summer camp. Dennis does not transform into a vampire by Drac’s stunts; but Dennis grows his fangs quickly and his vampire abilities demonstrate immediately, when Bela, the bat-like servant of Drac’s father Vlad, injures Winnie, who is monster Wayne’s daughter and has a crush on Dennis, and threatens to destroy Hotel Transylvania. Dennis begins to fight Bela with his sprouting superpower and fierce strength triggered by the close bonding between Winnie and himself. Drac, Mavis, Vlad, and other monsters soon join Dennis and defeat Bela and his giant-bat minions. Both Drac and Mavis grasp the meaning of education; Dennis achieves having vampire abilities and being true himself, while Johnny and Mavis decide to continue raising him in Transylvania.

Niemiec and Schulenberg (2011) also extended their research on integrating movies and positive psychology to understand death attitudes, in order to manage death anxiety with positive interventions. Using the compound word *cinematherapy*, Niemiec (2020) recently concluded as: “Character strengths cinematherapy offers clients an opportunity to look within, using a medium that is, at its best, enjoyable, engaging, and empowering. Strengths-spotting practices in the cinema can be a focal point in therapeutic discussions and serve as springboards for positive interventions.”

According to Hofstede (2001), there is often a shared perception within a society, a social environment that determines how individuals behave and interact with each other. The significant dimensions of cultural values to differentiate cultural differences are whether societies are individualistic or collectivistic and whether they have strong or weak uncertainty avoidance. Collectivistic cultures stressed the importance of interdependence and uncertainties are avoided in high uncertainty culture (Lee et al., 2021), i.e., what is accepted in the United States might be different from what is accepted in Korea, hence movie acceptance and influence may vary across different cultures. However, Hofstede approach has been criticized for its data collection method and outdated data (Eringa et al., 2015), while Handford (2020) warns that his model may develop a stereotype image of others.

### Purpose of the Study

This study investigates the wellbeing associated with learning the meanings of being different and growing in emotional resilience, flexibility, and openness to other cultures through movies.

Therefore, in this research, we want to answer the question: What is the extent to which films can be a good support in developing intercultural competences and increasing wellbeing?

The specific research questions are as:

1. Are there relationships between the level of intercultural competence, cultural intelligence, and the level of wellbeing?

2. Are there differences between the two groups of participants, based on cultural differences, in the level of development of intercultural competence, level of cultural intelligence, and level of wellbeing?

3. What is the effectiveness of the intervention program on intercultural competence, cultural intelligence, and level of wellbeing?

4. What are the participants’ opinions on the influence of cinema on intercultural education?

### Participants and Data Collection

The group of participants is composed of 142 (60.2%) students from Romania, Transilvania University of Brasov and 94 (39.8%) students from Universiti Brunei Darussalam. Initially, a group of 300 participants was recruited with an equal number of students from both universities. Only complete responses were kept in the analysis. Students were rewarded for their participation in this study by being recognized for 2h of internship activities. The participant group consists of 173 females (73.3% of the total), 59 males (25%), and four individuals (1.7%) did not wish to declare their affiliation. The participants are aged between 18 and 49years, with a mean of 20.45 (standard deviation 2.76). The ethnicity of the Romanian students is Romanian (60.2%), Hungarian (19.8%), German (8%), Roma (7%), and a few declared themselves as having two ethnicities, Romanian and Hungarian (2%), Romanian and Roma (2%), and Romanian and German (1%). Students from Brunei are of Bruneian (70%), Bruneian and Malay (20%), Chinese (4%), Chinese and Thai (3%), and Dusun (2%), Bruneian and Filipino (1%) ethnicity. The predominant religion in the Romanian student group is Christianity and Islam in the Brunei student group. Buddhism is also represented in a very small proportion. The majority of students (74%) has traveled abroad at least once and 26% have never been abroad. They are students on similar degree programs. All students are from the field of socio-human studies (educational sciences, sociology, communication, and arts).

### Data Analysis

This study was based on an experimental research design, with two parallel, relatively equivalent groups (one in Romania and the other in Brunei), with two measurement moments, pretest and posttest, within-subject, and between-subjects.

The following instruments were used at the pretest time: Butler and Kern’s PERMA-Profiler (2016), Cultural Intelligence Scale (Van Dyne et al., 2012); Intercultural Competence Inventory (Brinkman and Wink, 2007). In the posttest, the same instruments were used as in the previous stage and another instrument was used to address the influence of film on intercultural education (Popa, Nechita, Liu and Chin, 2021).

The experimental intervention consisted of viewing two films, *EPL* (Murphy, 2010) and *HT2* (Tartakovsky, 2015), and subsequent group discussions about the movies’ respective messages. Students were divided into small, conventional groups (due to the COVID 19 situation, it was only possible to work with 18–20 students in face-to-face activities). After watching each film, the students participated in a group discussion session of one hour each. The main topics of discussion were the themes of the films, the feelings they had while watching the films and afterward, the aspects they could add as learning experiences, about analogies with their personal lives. The films were analyzed from the perspective of cultural elements that become part of personal identity. The extent to which films become cultural products, that could influence any society in which they are shown, was also examined. Collective discussions were chosen in order not to embarrass shy students, and, to observe the syntality of groups.

Prior to enrollment in the research, participants were asked for informed consent. They were informed about the purpose of this study, research methods and instruments, and the intervention. They were also informed about their rights as research participants and data confidentiality issues. The research has received the approval of the Ethics Commission for Socio-Human Research of the Transilvania University of Brasov, Romania.

The instrument used for measuring students’ wellbeing is Butler and Kern’s PERMA-Profiler (2016). The instrument has 15 items, divided into five subscales: “Positive emotion: valence and arousal for positive emotion; Engagement: absorption, interest, and involvement; Relationships: connection with others, satisfaction, and giving/receiving support; Meaning: sense of direction, transcendence (connecting to something bigger than oneself), and the sense of value/worth; Accomplishment: self-efficacy, sense of accomplishment, and achieving personal goals” (Butler and Kern, 2016, p. 5). Cronbach’s alpha coefficient values were between.69 and.90, demonstrating an acceptable level for further analysis. Although the authors of the instrument use a response scale from 0 to 10, we preferred a scale from 1 to 7, with 1 being the lowest and 7 the highest. The arguments in favor of this choice were the intention to facilitate the completion of the instruments.

The intercultural competence was measured by Cultural Intelligence Scale (Van Dyne et al., 2012) and Intercultural Competence Inventory (Brinkman and Wink, 2007). The Cultural Intelligence (CQ) Scale measures individuals’ abilities to function effectively in cross-cultural environments. The instrument has four subscales: cognitive, metacognitive, motivational, and behavioral dimensions (Ang et al., 2007) and a response scale from 1 to 7, where 1 represents strongly disagree and 7 strongly agree. The Cognitive CQ subscale consists of 12 items that test understanding of similarities and differences between cultures. The Metacognitive CQ subscale, with 9 items, refers to the process of acquiring culture-specific information and its influence on culture-specific experiences (Culture General Knowledge and Context-Specific Knowledge). The Motivational CQ subscale has nine items that test individuals’ intention to learn about other cultures and confidence in their own strengths to act effectively in culturally mixed environments. The Behavioral CQ subscale contains nine items reflecting the individual’s ability to select appropriate behaviors in environments different from the culture of origin. The CQ Scale contains 39 items. The scale was used by permission of the Cultural Intelligence Center.

Intercultural Competence Inventory (Brinkman and Wink, 2007) is a questionnaire of 27 statements which the respondents can answer on a 5-item scale, ranging from never to always. The instrument has four dimensions: the first investigates self-monitoring, adaptability, and flexibility, the second checks the individual’s ability to value people from different cultures, the third focuses on the individual’s ability to build relationships with others, and the last investigates behaviors related to stress management, listening, and observation skills. In this research, we used only the subscales Building relationships with other 10 items and the subscale Listening and observation skills with four items, in order not to overburden the participants and following the analysis of the results obtained by Brinkman and Wink (2007), the other subscales not having the expected results.

The opinion questionnaire investigating the influence of cinema on intercultural education (Popa, Nechita, Liu and Chin, 2021) contains 42 items with specific content related to the influences of films viewed by participants. The scale was reliable (α is 0.87), which allowed us to continue the research. The questionnaire has a 7 item section focusing on the influence of films in general (type of messages offered, film from the perspective of cultural products, interest formed, and impact on the audience’s views of other cultures). The second section with 17 items investigates the opinions formed after watching *EPL* (character characteristics, message conveyed, reflection of other cultures, stimulation of tourist interest, and stimulation of lifestyle adoption). The third section containing 18 items focuses on the opinions formed after watching *HT2* (cultural appreciation, reflecting on one’s own upbringing, encouraging intercultural experiences, negotiating, and respecting cultural norms).

The data were statistically analyzed using IBM SPSS Statistic 23. The statistical analyses used were correlational analyses, parametric t-tests for comparison of means.

## Results

The first research question aimed to investigate the relationships between the level of intercultural competence, cultural intelligence, and the level of wellbeing. Table 1 summarizes the Pearson correlation coefficients showing the existence of the correlations between PERMA and Cultural Intelligence dimensions. Even if the correlation coefficients have small and medium values, we note the existence of statistically significant and strongly statistically significant correlations between the Cognitive and Metacognitive CQ subscales and all PERMA model dimensions. Interestingly, there are no statistically significant correlations between the PERMA model dimensions and the Motivation and Behavioral subscales. It is likely that the intention to interact with other cultures and the level of confidence in one’s ability to manage the stress of interacting with people from different cultures, in the ability to adapt to living conditions different from those specific to the culture of origin are not associated with positive emotions experienced, having direction in life, sense of working toward and reaching goals, mastery, and efficacy to complete tasks. However, this aspect deserves further research.

**Table 1:** Correlations between PERMA and cultural intelligence dimensions.

	M	SD	1	2	3	4	5	6	7	8	9
1 Positive Emotion	4.98	1.07	–								
2 Engagement	5.36	0.96	0.40^**^	–							
3 Relationship	5.37	1.06	0.63^**^	0.40^**^	–						
4 Meaning	5.14	1.02	0.70^**^	0.44^**^	0.55^**^	–					
5 Accomplishment	4.82	1.18	0.64^**^	0.52^**^	0.46^**^	0.65^**^	–				
6 Motivation	5.70	0.77	0.06	−0.05	0.13	0.03	0.02	–			
7 Metacognitive	5.31	0.90	0.26^*^	0.20^**^	0.19^**^	0.21^*^	0.15^*^	0.27^**^	–		
8 Behavioral	4.90	1.07	−0.01	−0.05	−0.09	−0.01	−0.05	0.25^**^	0.41^**^	–	
9 Cognitive	4.21	0.94	0.24^*^	0.23^*^	0.21^*^	0.32^*^	0.17^*^	0.26^*^	0.33^*^	0.25^*^	–
Cronbach’s alpha			0.65	0.74	0.68	0.75	0.81	0.63	0.77	0.61	0.79

* *p<* 0.05;

** *p<* 0.01.

Table 2 synthesized those existing between PERMA and Intercultural Competence dimensions. We observe the existence of highly significant, inversely proportional, bidirectional correlations between the Listening, observation skills of Intercultural Competence dimensions, and the Engagement and Accomplishment subscales. The existence of these associations demonstrates that engaging in behaviors that involve selecting effective communication strategies interrupts the state of flow, the focus on the task, being more concerned with appearances. Also, increased attention to listening, observation skills diminishes sense of working toward and reaching goals, mastery, and efficacy to complete tasks. Table 3 shows the existence of strongly significant positive bidirectional correlations between Cultural Intelligence and Intercultural Competence dimensions. These associations were expected, the only interesting aspect to investigate further is related to the values of the correlation coefficients which are small and moderate. This shows us that the strength of the linear relationship between the two variables is not very large. We expect the coefficients to be higher due to an overlap between the two investigated dimensions reported in the literature (Li, 2020).

**Table 2:** Correlations between PERMA and intercultural competence dimensions.

	M	SD	1	2	3	4	5	10	11
1 Positive Emotion	4.98	1.07	–						
2 Engagement	5.36	0.96	0.40^**^	–					
3 Relationship	5.37	1.06	0.63^**^	0.40^**^	–				
4 Meaning	5.14	1.02	0.70^**^	0.44^**^	0.55^**^	–			
5 Accomplishment	4.82	1.18	0.64^**^	0.52^**^	0.46^**^	0.65^**^	–		
10 Listening, observation skills	3.30	0.73	−0.10	−0.31^**^	−0.10	−0.03	−0.19^**^	–	
11 Building relationships	4.09	0.59	0.31^**^	0.40^**^	0.27^**^	0.28^**^	0.35^**^	0.15	–
Cronbach’s alpha			0.65	0.74	0.68	0.75	0.81	0.76	0.82

^*^p< 0.05;

** *p<* 0.01.

**Table 3:** Correlations between cultural intelligence and intercultural competence dimensions.

	M	SD	6	7	8	9	10	11
6 Motivation	5.70	0.77	–					
7 Metacognitive	5.31	0.90	0.26^**^	–				
8 Behavioral	4.90	1.07	0.25^**^	0.41^**^	–			
9 Cognitive	4.21	0.94	0.26^**^	0.33^**^	0.25^**^	–		
10 Listening and observation skills	3.30	0.73	0.19	0.53^**^	0.27^**^	0.21^**^	–	
11 Building relationships	4.09	0.59	0.33^**^	0.22^**^	0.25^**^	0.26^**^	0.15	
Cronbach’s alpha			0.63	0.77	0.61	0.79	0.76	0.82

^*^p< 0.05;

** *p<* 0.01.

The second research question targeted the existence of possible differences between the two groups of participants, based on cultural differences, in the level of development of intercultural competence, level of intercultural intelligence, and level of wellbeing. The results show that there are highly significant positive differences between the means of the two groups in the level of wellbeing self-reported. Thus, students from Brunei report higher levels of wellbeing compared to Romanian students (Table 4). There are no statistically significant differences between the groups on the following CQ subscales: Motivation, Metacognitive, and Cognitive. At the level of the Behavioral CQ and Intercultural competence subscales, there are statistically significant negative differences. Thus, the group of Romanian students reports higher levels of intercultural competence development and pay more attention to formal aspects of encounters with other cultures.

**Table 4:** Nationality differences for PERMA wellbeing, cultural intelligence, and cultural competence dimensions.

		N	M	SD	Difference	t	Effect size (Cohen’ d)
PERMA Positive Emotion	Romanian	142	5.28	1.16	0.75	5.63^***^	0.73
	Bruneian	94	4.54	0.71			
PERMA Engagement	Romanian	142	5.80	0.84	1.09	10.29^***^	1.30
	Bruneian	94	4.71	0.73			
PERMA Relationship	Romanian	142	5.54	1.18	0.41	2.96^**^	0.38
	Bruneian	94	5.13	0.80			
PERMA Meaning	Romanian	142	5.32	1.14	0.45	3.42^***^	0.45
	Bruneian	94	4.87	0.71			
PERMA Accomplishment	Romanian	142	5.32	1.11	1.25	9.29^***^	1.21
	Bruneian	94	4.07	0.85			
CQ Motivation	Romanian	142	5.67	0.87	−0.02	−0.15	0.02
	Bruneian	94	5.69	0.75			
CQ Metacognitive	Romanian	142	5.23	0.97	−0.21	−1.78	0.23
	Bruneian	94	5.44	0.77			
CQ Behavioral	Romanian	142	4.77	1.13	−0.29	−2.10^*^	0.27
	Bruneian	94	5.07	0.95			
CQ Cognitive	Romanian	142	4.26	0.94	0.12	1.03	0.13
	Bruneian	94	4.13	0.93			
Intercultural competence	Romanian	142	3.06	0.73	−0.62	−7.00^***^	0.91
	Bruneian	94	3.68	0.56			

* *p<* 0.05;

** *p<* 0.01;

*** *p<* 0.001.

The third research question concerned the effectiveness of the intervention program on intercultural competence, cultural intelligence, and level of wellbeing. As the intervention program was minimal, the changes are not very large either. However, it is gratifying that the viewing of the two films and the non-formal discussions after the viewing resulted in outcomes showing an increase in the level of positive emotions felt, a higher level of engagement and awareness (Metacognitive CQ) of how cultural background influences the interactions with people from different cultures (Table 5).

**Table 5:** Paired samples test differences for wellbeing and cultural intelligence.

		M	SD	Difference	t
PERMA Positive Emotion	T1	4.98	1.07	0.60	20.56^***^
	T2	5.59	0.91		
PERMA Engagement	T1	5.36	0.96	0.70	24.56^***^
	T2	6.06	0.78		
CQ Awareness	T1	5.64	1.03	−0.70	17.94^***^
	T2	6.34	0.67		

*N*= 236,

*** *p<* 0.001.

One last research question addressed the participants’ opinions on the influence of cinema on intercultural education. The instrument constructed by the authors of the research has a Cronbach’s Alpha coefficient of.97, which allows for further study. The vast majority of participants (60.8%) believe that films have the capacity to reach mass audiences globally. A high proportion of students (58.3%) believe that films provide powerful persuasive messages, letting people learn through imagination and play. Thus, films with cultural values can deliver results in line with the objectives that film producers set themselves. Participants (62.3%) believe that the effect of globalization on the film industry makes films cultural products that could influence any society in which they are shown. Respondents (69.6%) perceive films as a means to learn about different cultures and to observe and understand cultural differences in a positive way. The most important feature of the main character of the *EPL* film is her incredible power to live, more than half of the participants (53.9%) said that this character inspires them to fight for the life they want to live. Some respondents (52.5%) said that as a result of this film, they felt inspired to stay strong and fight against possible depression. The film stimulated the desire for freedom in lifestyle for 67.2% of participants. 54.9% of students said that the *EPL* film accurately reflected different cultures through narratives and characters. The film also inspired 55.9% of students to visit Italy, India, and the island of Bali. Interest in other cultures was stimulated for 66.2% of respondents. *EPL* provided moments of reflection on life for 73.2% of participants and encouragement to step out of their comfort zone for 55.4% of students.

*HT2* was a film reflecting different cultural perspectives for 65.7% of participants. Interest in visiting Transylvania (Romania) and California (United States) was stimulated for 55.4% of students. The vast majority of participants reflected on their own education after this film. They wanted to experience life in different cultural environments 59.3% of the respondents. *HT2* encouraged 66.7% of participants to learn about other cultures and to look at the world with a more open mind. A good proportion (74.6%) felt that the two educational typologies compared increased the fun effects. From the *HT2* perspective, 59.8% students stated that inclusivity of different cultural codes and viewpoints is essential for film culture and honest presentation of a richer narrative. The vast majority (67.1%) believed that the happy ending of *HT2* involves negotiating and respecting two cultures toward each other.

## Discussion

The aim of this research was to investigate the extent to which films can be used as support in the development of intercultural competences and in increasing wellbeing level. The study confirmed that films can be helpful in the development of intercultural competences. Participants expressed a higher level of awareness (Metacognitive CQ) of how cultural background influences the interactions with people from different cultures (*t*=17.94, *p*<0.001). These results confirm the findings of recent research in this direction (Truong and Tran, 2014; Cao and Meng, 2020; Nematzadeh et al., 2021). Many studies that have used films in pedagogical interventions have rather focused on improving language skills, but in addition to the main purpose, higher cultural awareness (Peng and Wu, 2016; Chaya and Inpin, 2020) and a higher level of tolerance for ambiguity and differences have often been achieved (Genç, 2018).

Research results also showed that participants achieved higher levels of engagement (*t*=24.56, *p*<0.001) and positive emotion (*t*=20.56, *p*<0.001) following the intervention. The results are similar to those obtained by Cao and Meng (2020), who highlighted that the use of foreign films and TV shows in psychoeducational interventions facilitates the reduction of participants’ anxiety when anticipating and engaging in outgroup interactions and generates positive emotions, such as comfort and ease of interacting with people from other cultures. Other studies show that films can be successfully used systematically in the teaching – learning process to improve positive traits and behaviors (Smithikrai, 2016) and increase wellbeing (Niemiec, 2014).

This comparative study has practical contribution to the field of positive psychology research. The individual reflections and group discussion of the two films helped develop their cultural competence and improve their wellbeing. The power of the group, the fact that opinions can be discussed in groups, the atmosphere that is created in the environment where the analysis takes place, leads to a much higher level of awareness compared to other settings where this was not explicitly foreseen.

The results confirm the existence of correlations between the variables. Instruments measuring Cultural Intelligence and Intercultural Competence have been used for the first time on the Romanian and Bruneian population. Furthermore, the results of the internal consistency coefficients of the scales are similar to those obtained with other studies (Brinkman and Wink, 2007; Li, 2020). As findings of Brinkman and Wink (2007) revealed that it is possible to train the intercultural competence of students, our study explored the role of movies in this direction. However, the results of the study showed that the use of films to develop intercultural competence has limited influence.

We follow the direction created by Lopez et al. (2002) to encourage studies in social science and clinical practice to introduce positive psychology in a multicultural context. We believe, like Nelson (2009), that an educational approach based on creating learning contexts that facilitate positive emotions can generate a higher level of cognitive flexibility, a greater degree of openness to addressing less familiar issues, and a greater degree of tolerance for cultural differences. This study also has some practical implications by presenting how using movies in educational contexts can enhance and develop both cultural intelligence and intercultural competence of the students as universities aim to involve them in internationalization projects. The bridges between students can be built with some 21st century tools that require digital, global, and visual skills as it is known that they prefer visuals to text rather than the opposite (Karkoulia, 2016). As other studies explored the relationship between cultural intelligence and wellbeing (Ayoob et al., 2015; Chen, 2015; Mehra and Tung, 2017) and intercultural competence and wellbeing (Wang et al., 2020), the present study contributed by exploring the relationships between the level of intercultural competence, cultural intelligence, and the level of wellbeing based on two samples of students from Romania and Brunei and using two well-known movies as a platform for shared understanding.

## Conclusion

This research investigated the role of films in developing intercultural competence and increasing wellbeing. An experimental design was used with two parallel, relatively equivalent groups (one in Romania and the other in Brunei), with two measurement moments, pretest and posttest, within-subject, and between-subjects. The results are not spectacular, but they are significant. The effectiveness of the intervention program on intercultural competence, cultural intelligence, and level of wellbeing has been demonstrated. The viewing of the two films (EPL and HT) and the non-formal discussions after the viewing resulted in an increase in the level of positive emotions felt, a higher level of engagement and awareness of the ways in which cultural background affects interactions with individuals from cultures different from one’s own.

The results are similar to other research. For example, Chao (2013) found that by using foreign movies, the students involved in an intercultural setting made real progress in developing intercultural motivation, attitudes, knowledge, and awareness. That is why we choose the two movies, *Eat Pray Love* (Murphy, 2010) and *Hotel Transylvania 2* (Tartakovsky, 2015), as our research cases. They are both Hollywood blockbusters and set in multiple locations around the world telling stories that focused on intercultural communication and interaction. Moreover, as aforementioned, the two chosen movies, albeit in quite differing ways, both manifest the respective characters’ practical passage through the six stages, i.e., denial, defense, minimization, acceptance, adaptation, and integration, in (DMIS) summarized by Bennett (2015, p. 519-524). The plot designs and, especially, the happy endings in both movies stress the distinctive cross-cultural value orientations, e.g., harmony, embeddedness, affective autonomy, and egalitarianism in the cultural value dimensions examined by Schwartz (2006). Therefore, the unequivocal messages conveyed in *EPL* (Murphy, 2010) and *HT2* (Tartakovsky, 2015) make them become two quintessential examples for our crosscultural studies on intercultural sensitivity and competence carried out in two representative universities located in eastern Europe and southeast Asia, respectively.

Typical pandemic conditions did not allow to conduct the experimental intervention as originally designed. Therefore, this aspect brought some constraints to the study. However, the results show that there were increases in wellbeing, a higher level of engagement and awareness. The concept of intercultural competence is based on valuing diversity; therefore, a value cannot be adopted after a short intervention. But the fact that there were increases in the engagement and awareness subscales shows that the participants employed in a reflective process, analyzing their own orientations, and opening to other cultures. All this occurred with an increased level of positive emotions.

The fact that self-reporting instruments were used gives the study certain specific limitations (social desirability). Measures have been taken to mitigate this possible effect. Research design can be improved by incorporating qualitative research methods. Future studies may investigate the mediating or moderating effects of wellbeing on the development of intercultural competence.

The future studies can explore the relationship between intercultural competence, cultural intelligence, and wellbeing by evaluating the common projects and interactive models of teaching and learning like COIL^1^ developed by Transilvania University of Brasov and University Brunei Darussalam. These projects will be further developed during and after COVID-19 pandemic and acknowledging that international and intercultural competency are underscored in a limited number of settings (de Wit and Altbach, 2021). Future intervention programs can also be improved.

## Data Availability Statement

The original contributions presented in the study are included in the article/supplementary material, further inquiries can be directed to the corresponding author.

## Ethics Statement

The studies involving human participants were reviewed and approved by Commission for Ethics in Social-Human Scientific Research, Transilvania University of Brasov. The patients/participants provided their written informed consent to participate in this study.

## Author Contributions

DP has the first authorship, FN is the correspondence author, and YL and SW share senior authorship. All authors contributed to the article and approved the submitted version.

## Funding

Funds received for open access publication fees, from our institution.

## Conflict of Interest

The authors declare that the research was conducted in the absence of any commercial or financial relationships that could be construed as a potential conflict of interest.

## Publisher’s Note

All claims expressed in this article are solely those of the authors and do not necessarily represent those of their affiliated organizations, or those of the publisher, the editors and the reviewers. Any product that may be evaluated in this article, or claim that may be made by its manufacturer, is not guaranteed or endorsed by the publisher.

## Footnotes

^1^ http://http://coil.suny.edu/
